# Activity-based protein profiling of the hepatitis C virus replication in Huh-7 hepatoma cells using a non-directed active site probe

**DOI:** 10.1186/1477-5956-8-5

**Published:** 2010-02-04

**Authors:** Ragunath Singaravelu, David R Blais, Craig S McKay, John Paul Pezacki

**Affiliations:** 1 Steacie Institute for Molecular Sciences, National Research Council Canada, Ottawa, Ontario, K1A 0R6, Canada; 2 Department of Biochemistry, Microbiology and Immunology, University of Ottawa, Ottawa, Ontario, K1N 6N5, Canada; 3 Department of Chemistry, University of Ottawa, Ottawa, Ontario, K1N 6N5, Canada

## Abstract

**Background:**

Hepatitis C virus (HCV) poses a growing threat to global health as it often leads to serious liver diseases and is one of the primary causes for liver transplantation. Currently, no vaccines are available to prevent HCV infection and clinical treatments have limited success. Since HCV has a small proteome, it relies on many host cell proteins to complete its life cycle. In this study, we used a non-directed phenyl sulfonate ester probe (PS4≡) to selectively target a broad range of enzyme families that show differential activity during HCV replication in Huh-7 cells.

**Results:**

The PS4≡ probe successfully targeted 19 active proteins in nine distinct protein families, some that were predominantly labeled *in situ *compared to the *in vitro *labeled cell homogenate. Nine proteins revealed altered activity levels during HCV replication. Some candidates identified, such as heat shock 70 kDa protein 8 (or HSP70 cognate), have been shown to influence viral release and abundance of cellular lipid droplets. Other differentially active PS4≡ targets, such as electron transfer flavoprotein alpha, protein disulfide isomerase A5, and nuclear distribution gene C homolog, constitute novel proteins that potentially mediate HCV propagation.

**Conclusions:**

These findings demonstrate the practicality and versatility of non-directed activity-based protein profiling (ABPP) to complement directed methods and accelerate the discovery of altered protein activities associated with pathological states such as HCV replication. Collectively, these results highlight the ability of *in situ *ABPP approaches to facilitate the identification of enzymes that are either predominantly or exclusively labeled in living cells. Several of these differentially active enzymes represent possible HCV-host interactions that could be targeted for diagnostic or therapeutic purposes.

## Background

**Figure 1:**
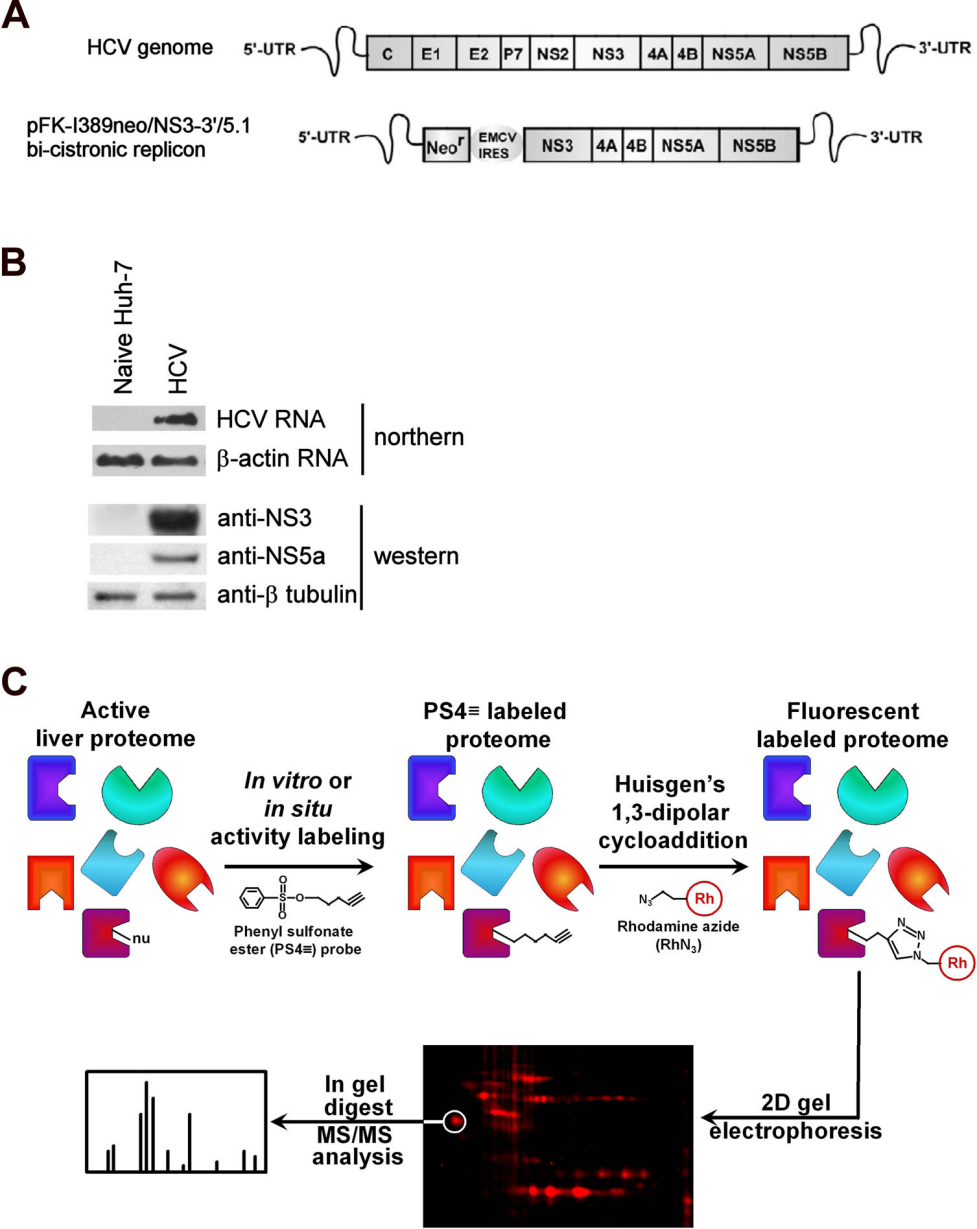
**Structure and protocol of the PS4≡ probe used *in vitro *and *in situ *to target the active Huh-7 proteome during HCV replication**. (A) Schematic representations of the HCV genome and the bi-cistronic (pFK-I389neo/NS3-3'/5.1) subgenomic replicon used in this study (NS, non-structural proteins). (B) Northern analysis of HCV and β-actin (as a loading control) RNA (upper two blots) and detection of HCV NS3, HCV NS5A, and β-tubulin (as a loading control) proteins by western blotting (lower three blots) in naïve Huh-7 human hepatoma cell lines and in Huh-7 cells stably replicating HCV (HCV). (C) Scheme of the ABPP protocol used to identify phenyl sulfonate ester (PS4≡) targets. The active proteome of naïve Huh-7 cells (not shown) or Huh-7 stably expressing the HCV replicon was labeled through a nucleophilic (Nu) residue within the active site of the enzyme targeted by the reactive group of the PS4≡ probe. After isolation, the labeled proteome was conjugated with the rhodamine azide reporter tag (RhN_3_) via the Huisgen's 1,3-dipolar cycloaddition and separated by two-dimensional gel electrophoresis. The fluorescently tagged proteins were identified by LC-MS/MS.

The hepatitis C virus (HCV) is the major causative agent of hepatitis that affects over three percent of the global population [1]. With no vaccines yet available and clinical treatments that have only limited success, many HCV-infected individuals develop chronic hepatitis which eventually progresses into liver steatosis, cirrhosis and hepatocellular carcinoma. HCV is the leading cause of liver disease and transplantation [2]. As the HCV genome is a single-stranded RNA molecule of only ~9.6 kb (Figure 1A) that encodes for ten mature viral proteins, HCV relies heavily on host factors for its propagation. During cell entry, HCV E1 and E2 viral proteins interact with four known host receptors CD81, claudin-1, SRB1, and occludin [3]. Unpackaging is followed by the synthesis of the HCV polypeptide by host cell ribosomes that is subsequently processed into ten viral proteins by host cellular signal peptidase, signal peptide peptidase, and viral proteases NS2 and NS3/4A [4]. Host-mediated post-translational modifications, such as glycosylation of E1 and E2 [5], phosphorylation of NS5A [6], as well as the direct interaction with host cell geranylgeranylated FBL2 protein [7] and liver microRNA miR-122 [8,9], are also deemed essential for viral protein maturation and proper viral infectivity.

High throughput techniques, such as gene expression profiling and proteomic approaches have led to the identification of several host-viral interactions [10,11]. However, some host cell factors involved in HCV propagation are likely regulated both by post-translational modifications and association with cofactors and regulatory proteins, for which conventional abundance-based genomic and proteomic techniques provide no direct information. Activity-based protein profiling (ABPP) was developed to provide a direct insight into changes in catalytic activity of enzyme classes in complex proteomes [12-14] and to annotate previously unknown enzymatic functions of proteins [15]. ABPP employs active site-directed covalent probes that consist of small molecule inhibitors linked to reporter tags [12] that exploit conserved mechanistic features of their target enzyme superfamily. Examples of ABPP probes includes fluorophosphonates, acyloxymethyl ketones, and amino acid coupled quinolimine methides that target serine hydrolases [16] and proteases [17,18]. We have previously applied some of these directed probes to study the differential activity of host cell proteases during HCV replication [19].

Although directed ABPP is effective for targeting enzymes with known covalent inhibitors, there is a lack of such cognate inhibitors for many enzyme classes. To extend the number of enzyme classes addressable by ABPP, non-directed probes were designed through the screening of large libraries of small molecule inhibitors against complex proteomes for activity-dependent protein reactivity [20,21]. Non-directed sulfonate ester [20], α-chloroacetamide [22], and spiroepoxide [23] probes were successfully used to target a wider range of protein candidates through their reactivity with numerous enzyme families. Herein, we applied activity-based profiling using a non-directed phenyl sulfonate ester (PS4≡) probe [20] to examine a wider range of enzyme families involved in HCV replication.

## Results and discussion

### Characterization of non-directed activity based labeling

Directed ABPP probes have successfully targeted over a dozen of different enzyme classes [24,25]. However, due to the lack of cognate affinity labels, numerous enzymes remain beyond the reach of directed ABPP. In this study, we investigated the ability of ABPP approaches using the non-directed PS4≡ probe to profile enzyme activity in Huh-7 cells harboring the genotype 1b HCV subgenomic replicon RNA (Figure 1) [26,27]. The selection of the PS4≡ probe was based on its activity-based reactivity towards a large but manageable fraction of the proteome (over half a dozen distinct enzyme families are known to be targeted) [21,28] and on its relatively small size, which should minimize non-covalent probe-protein interactions and allow cellular uptake and distribution for *in situ *labeling.

**Figure 2:**
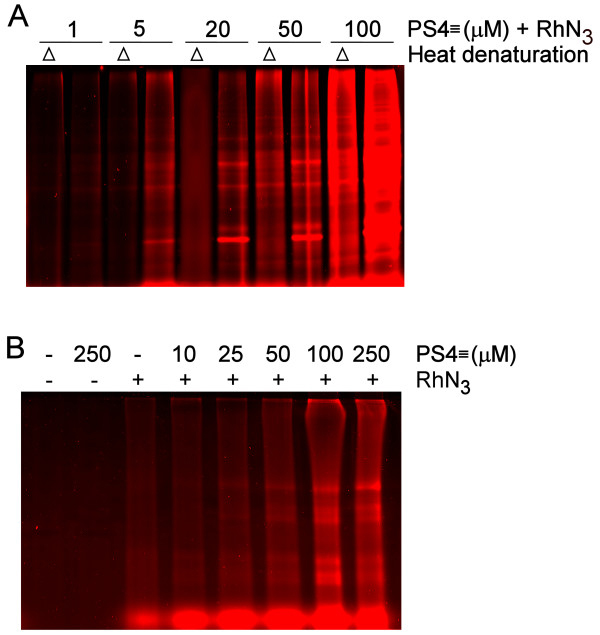
***In vitro *and *in situ *PS4≡ probe condition optimization for labeling the active proteomes from Huh-7 cells**. (A) Active proteomes isolated from naïve Huh-7 cells were treated at 37°C for 60 min with various concentrations of substrate based probes (PS4≡) either with or without a denaturing preheating step (Δ = 100°C, 5 min), subjected to click chemistry with RhN_3_, separated by SDS/PAGE electrophoresis, and visualize by in-gel fluorescence scanning. The *in vitro *optimal PS4≡ concentration with the strongest signal from the active proteome and least non-specific binding from the denatured proteome was determined to be 20 μM. (B) *In situ *optimal labeling conditions for the naïve Huh-7 proteome to give the strong and similar banding patterns as experiments conducted *in vitro *was determined to be an hour incubation of 100 μM of PS4≡ probe in cell culture media. Non-specific fluorescence was observed to be negligible when either the PS4≡ probe or RhN_3 _was omitted.

To achieve in-gel fluorescence detection of the labeled protein candidates, a rhodamine dye was coupled to an azide group for a subsequent conjugation via Huisgen's 1,3-dipolar cycloaddition ('click' chemistry) with the alkyne group on the probe (Figure 1C). The configuration of both bioorthogonal coupling partners, the alkyne on PS4≡ and the azide on the rhodamine reporter (RhN_3_), was chosen over the reverse position of functional groups of the cycloaddition reaction (i.e. PSN_3 _and Rh≡) as the first configuration proved to significantly minimize non-specific labeling of abundant proteins [28] (Figure 2).

**Figure 3:**
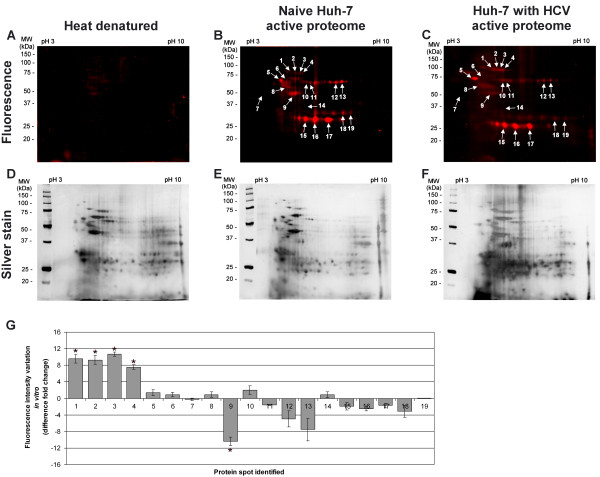
**PS4≡ protein profiling is activity specific in hepatoma Huh-7 cells**. The active proteome (200 μg) isolated from naïve Huh-7 cells (B) and Huh-7 cells stably replicating HCV (C) was labeled *in vitro *for 1 h at 37°C with 20 μM of PS4≡ probe. A representative gel of the denaturing preheating step of 100°C for 5 min (Δ) was performed as a control (A) to confirm the PS4≡ probe specificity toward active enzymes. After separation of the proteomes by two-dimensional gel electrophoresis, the fluorescent-tagged proteins were scanned for fluorescence (A - C) and subjected to silver staining (D - F). The labeled proteins, indicated with arrows, were compared with *in situ *profiling (Figure 4) and sent for identification by LC-MS/MS (Table 1). The other unmarked fluorescent spots were considered non-specific background as they didn't meet the inclusion criteria of a MASCOT score above 50, a corresponding pI and molecular weight, as well as being identified in at least three independent experiments. (G) The differential activity of fluorescently-labeled proteins *in vitro *was quantified with the ImageJ software (NIH) by measuring the fluorescence intensity (normalized for volume) of the corresponding protein spot of HCV replicating Huh-7 cells (C) versus of that of naïve Huh-7 cells (B). Statistically significant differences were determined using ANOVA, with probabilities *p < 0.05.

The PS4≡ probe specificity was determined *in vitro *with the isolated proteome of Huh-7 cells resuspended in sodium phosphate buffer, for which the optimal labeling condition was observed to be 20 μM for one hour at 37°C (Figure 2A). At this concentration, the heat denatured proteome revealed minimal non-specific reactivity while displaying maximal sensitivity (Figures 2A, 3A-C). At first glance, the PS4≡ probe had a unique fluorescence labeling profile (Figure 3) when compared with amino acid coupled quinolimine methide probes in Huh-7 cells [19]. Five spots (Figure 3G, #1, 2, 3, 4, 9), comprising isomerases and chaperones (Table 1), displayed a significant differential activity *in vitro *between naïve Huh-7 cells and those stably replicating HCV, highlighting the importance of the PS4≡ non-specific probe to complement directed ABPP for providing a broader assessment of the differentially active proteome between control cells and cells replicating HCV RNA.

**Table 1:** Identification of labeled proteins with the PS probe from Figures 3 and 4.

Gel band*	Protein	Function	Accession no. (NCBI)	Mass (kDa)	pI	MASCOT score**	Sequence coverage (%)	Peptide sequence
1	Protein disulfide isomerase-associated 4	Isomerase	4758304	72.89	4.96	57	3.4	-FDVSGYPTIK -IDATSASVLASR

2	Heat shock 70 kDa protein 5	Chaperone	16507237	72.29	5.07	929	36.2	-VMEHFIK -VLEDSDLK -LTPEEIER -ITITNDQNR -DAGTIAGLNVMR -VEIIANDQGNR -DAGTIAGLNVMR + Ox. of M -FEELNMDLFR -NELESYAYSLK -ELEEIVQPIISK -TWNDPSVQQDIK -SDIDEIVLVGGSTR -TFAPEEISAMVLTK -TFAPEEISAMVLTK + Ox. of M -ITPSYVAFTPEGER -IINEPTAAAIAYGLDK -NQLTSNPENTVFDAK -SQIFSTASDNQPTVTIK -LYGSAGPPPTGEEDTAEKDEL

3	Heat shock 70 kDa protein 8 isoform 1	Chaperone	5729877	70.85	5.37	767	32.5	-ITITNDK -LSKEDIER -FELTGIPPAPR -DAGTIAGLNVLR -VEIIANDQGNR -YKAEDDLQR -MVNHFIAEFK -FEELNADLFR -NSLESYAFNMK + Ox. of M -SQIHDIVLVGGSTR -TTPSYVAFTDTER -SFYPEEVSSMVLTK -NQVAMNPTNTVFDAK -IINEPTAAAIAYGLDK -NQVAMNPTNTVFDAK + Ox. of M -STAGDTHLGGEDFDNR -TVTNAVVTVPAYFNDSQR

4	Heat shock 70 kDa protein 9	Chaperone	12653415	73.68	6.03	665	22.2	-VLENAEGAR -RYDDPEVQK -QAASSLQQASLK -DAGQISGLNVLR -VQQTVQDLFGR -AQFEGIVTDLIR -SDIGEVILVGGMTR -TTPSVVAFTADGER -LYSPSQIGAFVLMK -QAVTNPNNTFYATK -LLGQFTLIGIPPAPR -NAVITVPAYFNDSQR

Gel band*	**Protein**	**Function**	**Accession no. (NCBI)**	**Mass** **(kDa)**	**pI**	MASCOT score**	**Sequence coverage (%)**	**Peptide sequence**

5	Vimentin	Structural, filament	340219	53.68	5.03	487	24.5	-QVDQLTNDK -DNLAEDIMR -QDVDNASLAR -FADLSEAANR -VELQELNDR -EYQDLLNVK -ILLAELEQLK -LGDLYEEEMR -NLQEAEEWYK -SLYASSPGGVYATR -ISLPLPNFSSLNLR

6	Prolyl 4-hydroxylase, beta subunit precursor (Protein disulfide isomerase)	Isomerase (chaperone)	20070125	57.08	4.76	619	28.5	- FFPASADR - QLAPIWDK - ENLLDFIK - LKAEGSEIR - VHSFPTLK - ILEFFGLK - THILLFLPK - EADDIVNWLK - LITLEEEMTK - NFEDVAFDEK - MDSTANEVEAVK + Ox. of M - YQLDKDGVVLFK - VDATEESDLAQQYGVR - HNQLPLVIEFTEQTAPK

7	Alpha 2-HS-glycoprotein	Ion binding	156523970	39.33	5.43	136	6.3	-HTLNQIDEVK -CDSSPDSAEDVRK

8	ATP synthase beta subunit	Hydrolase	1374715	51.17	4.92	541	32.6	-IGLFGGAGVGK -IPVGPETLGR -VVDLLAPYAK -TIAMDGTEGLVR -TIAMDGTEGLVR + Ox. of M -IMNVIGEPIDER -IMNVIGEPIDER + Ox. of M -FTQAGSEVSALLGR -TVLIMELINNVAK -VALVYGQMNEPPGAR -VALVYGQMNEPPGAR + Ox. of M -AIAELGIYPAVDPLDSTSR

9	Protein disulfide isomerase A5	Isomerase	1710248	46.17	4.95	357	15.6	-AATALKDVVK -GESPVDYDGGR -TGEAIVDAALSALR -NLEPEWAAAASEVK -LAAVDATVNQVLASR -GSTAPVGGGAFPTIVER

10	Quinolinate phosphoribosyl-transferase	Transferase	13477197	30.81	5.82	109	11.4	-YDLGGLVMVK -DNHVVAAGGVEK -YGLLVGGAASHR

11	Nucleobindin	Ion binding	189308	53.70	5.15	62	2.8	-LVTLEEFLASTQR

12	Phosphoglycerate dehydrogenase	Oxidoreductase	5771523	56.63	6.29	196	10.5	- TLGILGLGR - GGIVDEGALLR - VTADVINAAEK - ILQDGGLQVVEK - GTIQVITQGTSLK

Gel band*	**Protein**	**Function**	**Accession no. (NCBI)**	**Mass** **(kDa)**	**pI**	MASCOT score**	**Sequence coverage (%)**	**Peptide sequence**

13	Aldehyde dehydrogenase 1	Oxidoreductase	2183299	54.80	6.30	293	11.0	- ILDLIESGK - VAFTGSTEVGK - QAFQIGSPWR - EEIFGPVQQIMK + oxid(M) - IFVEESIYDEFVR

14	Nuclear distribution gene C homolog	Protein binding	5729953	38.22	5.27	87	6.0	-SETSGPQIK -LVSSDPEINTK

15	Cytokine induced protein 29 kDa	Nucleotide, protein binding	32129199	23.66	6.10	66	9.0	-FGISSVPTK -ITSEIPQTER

16	Endoplasmic reticulum protein 29 isoform 1	Chaperone	5803013	28.97	6.77	269	25.7	- QGQDNLSSVK - SLNILTAFQK - FDTQYPYGEK - ESYPVFYLFR - GALPLDTVTFYK - DGDFENPVPYTGAVK

17	Proteasome subunit alpha type-1	Hydrolase	190447	30.21	6.51	143	10.6	-LVSLIGSK -ILHVDNHIGISIAGLTADAR

18	Purine nucleoside phosphorylase	Transferase	230387	32.13	6.45	84	9.7	-FEVGDIMLIR -LGADAVGMSTVPEVIVAR

19	Electron transfer flavoprotein, alpha polypeptide	Electron transport	4503607	35.06	8.62	156	16.5	-SPDTFVR -LEVAPISDIIAIK -GLLPEELTPLILATQK -AAVDAGFVPNDMQVGQTGK

**Figure 4:**
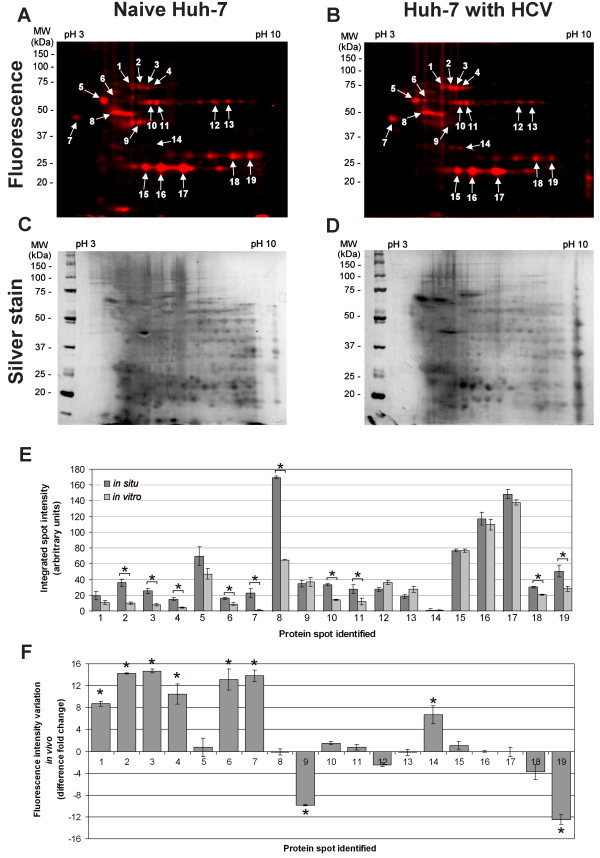
***In situ *PS4≡ protein profiling during HCV replication identifies nine differentially active proteins**. The PS4≡ probe was added to the cellular media of naïve Huh-7 cells (A) or Huh-7 stably expressing the HCV replicon (B) for 1 h at 37°C. The labeled proteome was isolated and subjected to Huisgen's 1,3-dipolar cycloaddition with rhodamine azide. After separation of the proteomes by two-dimensional gel electrophoresis, the fluorescently tagged proteins were scanned (A, B), silver stained (C, D), and identified by LC-MS/MS (Table 1). (E) The differential activity between *in situ *(Figure 4) and *in vitro *(Figure 3) fluorescently labeled proteins in Huh-7 cells was quantified with the ImageJ software (NIH) by measuring the fluorescence intensity (normalized for volume) of the corresponding protein spot. (F) The differential activity of fluorescently labeled proteins between HCV and naïve Huh-7 cells was quantified with the ImageJ software (NIH) by measuring the fluorescence intensity (normalized for volume) of the corresponding protein spot of HCV replicating Huh-7 cells (B) versus of that of naïve Huh-7 cells (A). Statistically significant differences were determined using ANOVA, with probabilities *p < 0.05.

* PS4≡ probe labeled protein bands numbered in Figures 3 and 4.

** MASCOT minimal protein score = 50.

### *In situ *PS4≡ labeling increases the number of ABPP hits

An additional advantage of the PS4≡ probe is conferred by its small molecular size for *in situ *ABPP. This, in turn, allows the labeling of active enzymes in their native subcellular environment to provide the most physiologically relevant enzymatic profile. The *in situ *conditions were optimized by varying the PS4≡ concentration in the cellular media to obtain a fluorograph of similar banding pattern, fluorescence intensity, and minimal background as to the *in vitro *labeling. Under normalized labeling conditions (*in situ *labeling for 1 hour with 100 μM in cell culture media; *in vitro *labeling for 1 hour with 20 μM probe in 1 mg/mL soluble proteome), the *in situ *fluorescent labeling profile in Huh-7 cells (Figure 4) was found quantitatively comparable to the *in vitro *profile (Figure 3), as judged by the fluorescent spot intensity of both profiles (Figure 4E) and taking into consideration the silver stained gels as loading controls (Figures 3D-F and 4C-D, 200 μg of proteome loaded per gel). The increased concentration of PS4≡ required for *in situ *labeling could be attributed to cellular uptake as the probe is thought to enter the cell via passive diffusion.

Although some of the proteins were labeled equally *in vitro *and *in situ*, the latter resulted in some proteins that were predominantly labeled *in situ *when compared to the cell homogenate (Figure 4E, spots # 2, 3, 4, 6, 7, 8, 10, 11, 18 and 19), which validates previous observations [28]. These enzymes included: a chaperone (heat shock protein 70), one isomerase (protein disulfide isomerase A1), two ion-binding proteins (alpha 2-HS-glycoprotein and nucleobindin), one electron transport protein (electron transfer flavoprotein alpha), one hydrolase (ATP synthase beta), and two transferases (quinolinate phosphoribosyl-transferase and purine nucleoside phosphorylase) (Table 1). The decreased *in vitro *labeling of some proteins may be attributed to the homogenate obtained by physical disruption of the Huh-7 cells for *in vitro *ABPP, altering the concentration and subcellular distribution of endogenous inhibitors/activators and protein-protein interactions needed for enzymatic activity [29]. Such physiologically disruptive effects would explain the significant reduction in ABPP labeling *in vitro *for ATP synthase beta (Figure 4E, spot 8); moreover, membrane disruption through proteome isolation likely resulted in the loss of the proton gradient needed for the conformational change of the β-subunits of the transmembrane enzyme. This would prevent the subsequent release of the ATP product and significantly abolishing the enzyme's hydrolase activity [30]. The enhanced labeling of some protein targets *in situ *could also be attributed to the uneven subcellular distribution of the probe taken by cells, which could lead to the enrichment of the probe in certain cellular organelles and disproportionately enhance protein labeling [28]. However, this enrichment would be expected to be consistent and not a significant variable factor among *in situ *samples analyzed simultaneously (i.e. Figure 4A-B) making the PS4≡ probe suitable for interrogating enzyme activity in their native cellular environment.

### Identification of labeled polypeptides in Huh-7 cells

To identify the enzymes labeled by the PS4≡ probe, the combination of fluorescence 2D-gel electrophoresis (2D-GE) and tandem mass spectrometry was used as outlined in Figure 1C. Fluorescence 2D-GE was chosen over multidimensional protein identification technology (MudPIT) for its high throughput quantitative analysis and topological protein information, in spite of lower protein detection and higher signal overlap from co-migrating background proteins, which are two potential limiting factors for 2D-GE [14].

*In vitro *and *in situ *ABPP of the active proteome from naïve Huh-7 cells and cells stably replicating HCV, analyzed by 2D-GE and LC-MS/MS, revealed 19 reactive candidates whose labeling were abolished when subjected to *in vitro *pre-denaturation step (Δ) at 100°C for 5 min (Figure 3). These candidates were considered positive hits, providing that they had a MASCOT score above 50, a corresponding pI and mass during 2D-GE migration, and reproducible identification in at least three independent experiments (Table 1). Due to the lack of eligibility criteria or reproducible identification, the other unmarked fluorescent spots were considered as non-specific background or co-migrating contaminants and were not further analyzed (data not shown).

Aside from the pre-denaturation step (Figure 2A, 3A), the activity dependent PS4≡ labeling is supported by the presence of known phenyl sulfonate targets in the reactive candidates, such as aldehyde dehydrogenase 1, for which the labeling has been shown to be blocked by the inhibitor disulfiram [28] and the cofactor NAD^+ ^[21], which suggests activity-dependent probe reactivity and cofactor-binding selectivity of targeted enzymes. Furthermore, mass spectroscopy analyses confirmed the binding of the PS4≡ probe to the catalytic pocket of the enzymes. Mutations of those catalytic residues abolished more than 90% the PS4≡ labeling compared to their wild-type counterpart [27]. In a catalytic assay, the probe (i.e. irreversible inhibitor)-bound wild-type targeted enzyme demonstrated more than a 90% reduction in activity toward its endogenous substrate [27]. These inhibition-based experiments validate the active-state specificity of the PS4≡ probe labeling toward its protein targets.

### Characterization of the PS4≡ probe targets

**Figure 5:**
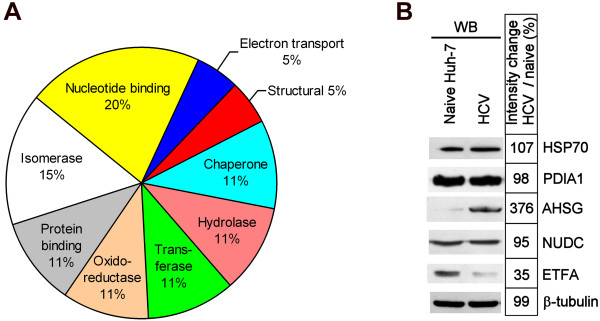
**PS4≡ non-directed activity-based profiling targets several protein classes during HCV replication**. (A) Compilation of the 19 true probe targets from Figures 3 and 4 and classification into their corresponding protein family (data from Table 1). (B) Expression analysis of five differentially active protein candidates: heat shock protein 70 kDa (HSP70), protein disulfide isomerase A1 (PDIA1), alpha 2-HS-glycoprotein (AHSG), nuclear distribution gene C homolog (NUDC), and electron transfer flavoprotein alpha polypeptide (ETFA) (Figure 4 and Table 1). The expression analysis was performed by western blotting in the naïve Huh-7 cell line and Huh-7 stably expressing the bi-cistronic pFK-I389neo/NS3-3'/5.1 subgenomic replicon (HCV).

The 19 PS4≡ probe targets were grouped into their respective protein families (Table 1, Figure 5A) where the majority of the candidates corresponded to enzyme classes previously labeled with phenyl sulfonate ester probes, including hydrolases, transferases, oxidoreductases, and isomerases [20,21,27,28,31]. Some of our Huh-7 PS4≡ hits were previously identified with phenyl sulfonate probes in human breast cancer cells and *in vivo *mouse models, such as aldehyde dehydrogenase 1 [20,27,28,32] and protein disulfide isomerase (i.e. prolyl 4-hydroxylase) [28], thereby validating our non-directed ABPP methodology in human hepatoma cells. The remaining candidates represent novel PS4≡ targets (Table 1). The variety of the PS4≡ hits (nine protein classes were identified in this study) (Figure 5A) could be reflected by the diversity of amino acids known to be labeled by the PS4≡ probe (aspartate, cysteine, glutamate, and tyrosine) [27], which reveal the versatility of the phenyl sulfonate reactive group in labeling a diversity of catalytic residues in a range of mechanistically distinct enzymes. Some proteins targeted in Huh-7 cells were shown to utilize these amino acids as catalytic residues in their active pocket. For example, protein disulfide isomerases (spots 1, 6 and 9) have cysteine catalytic residues [33] whereas phosphoglycerate dehydrogenase (spot 12), aldehyde dehydrogenase 1 (spot 13), and purine nucleoside phosphorylase (spot 18) possess a catalytic glutamate [34-36]. Both types of residues would likely be covalently labeled by the PS4≡ probe [27]. Alternatively, several previously PS4≡ labeled enzymes were shown not to utilize covalent catalysis [20,31], thus suggesting a second labeling mechanism where a non-catalytic residue (such as cysteine) in the active pocket of non-enzymatic hits could serve as the site of labeling by the PS4≡ probe [21]. The diversity of potential activity-based labeling mechanisms of the PS4≡ probe could explain the broad range of proteins identified in Huh-7 cells (Table 1, Figure 5A).

### *In situ *PS4≡ activity profiling during HCV replication

To assess the non-directed activity profile during HCV replication, PS4≡ profiling was performed on naïve Huh-7 cells and those stably replicating the pFK-I389neo/NS3-3'/5.1 subgenomic HCV replicon [26,37-42] at high levels [19]. Several PS4≡ targets (Table 1), including aldehyde dehydrogenase 1, protein disulfide isomerase, phosphoglycerate dehydrogenase, heat shock protein 70 kDa protein 5, ATP synthase beta subunit, and endoplasmic reticulum protein 29, were also detected with amino acid coupled quinolimine methide probes, confirming our previous findings [19] and supporting the concept that non-directed ABPP complements directed ABPP analysis through parallel [19] or multiplexed experiments [20]. Interestingly, HCV NS3/4A enzyme was not detected by the PS4≡ probe, even *in vivo*. Labeling experiments using a fluorophosphonate and several substrate-based ABPP probes on purified recombinant HCV NS3/4A showed reluctance of the enzyme towards ABPP labeling [19]. This lack of detection could be attributed to several factors, including the low abundance of NS3/4A relative to other host cell hydrolase or product inhibition of the enzyme [43].

Although heat shock 70 kDa protein 5 was also labeled by both the PS4≡ and amino acid coupled quinolimine methide probes *in vitro *[19], its activity was significantly higher during HCV replication *in situ *when compared to *in vitro *(Figure 4E, spot #2). The modest decrease in activity, although not completely abolished, of heat shock 70 kDa protein 5 after proteome extraction (Figure 4E, spot #2) suggests a potentially more important role for this protein during HCV replication. These observations further outline the benefits of *in situ *profiling of proteins in their native cellular environment to obtain their accurate activity in presence of their cellular endogenous inhibitors/activators.

To examine if the fluorescence intensity difference observed during HCV replication could solely be attributed to differential enzymatic activity or could reflect differences in gene expression, the expression levels of five protein candidates identified by non-directed ABPP during HCV replication were analyzed by western blotting (Figure 5B). Both AHSG and ETFA differential activity levels (Figure 4F) correlated with protein level variations (Figure 5B) between naïve Huh-7 cells and those stably replicating HCV thus suggesting that the expression level of these genes is altered in Huh-7 stably replicating HCV. The absence of any differential expression levels in the three remaining candidates (HSP70, PDIA1 and NUDC) could be explained by the fact that these enzymes display a differential activity during HCV replication, while having unchanged protein levels. Post-translational modifications or the HCV-mediated modulation of endogenous inhibitors/activators or subcellular localization could explain this observation.

Some PS4≡ identified candidates obtained from Huh-7 cells have been shown to be implicated in HCV or in HCV-related liver complications. For example, heat shock 70 kDa protein 8 (HSP70A8 or HSP70 cognate) was recently shown to associate with HCV core, E2 protein, and viral particles around lipid droplets (LDs) to influence viral release and the abundance of cellular LDs [44]. We recently showed LDs to be highly abundant in Huh-7 cells with high levels of HCV pFK-I389neo/NS3-3'/5.1 replication (unpublished observation), which could explain the higher HSP70A8 activity (Figure 4F, spot #3) resulting in more abundant LD and HSP70A8 aggregates [44]. Furthermore, the identified HSP70 proteins 5, 8 and 9, along with aldehyde dehydrogenase 1, vimentin, cytokine-induced protein 29 kDa, and electron transfer flavoprotein alpha (ETFA), are known markers and proteins of interest in HCV-associated hepatocellular carcinoma, cirrhosis and cellular interferon response [45-52].

The association of protein disulfide isomerase A1 and A4 activity (Figure 4, spots 1 and 6) with HCV replication levels was also observed with a novel origamicin probe [39], which reinforces the fact that these enzymes might catalyze cysteine oxidation and disulfide bond isomerization of HCV proteins, also known to be essential for the infectivity of other viruses (e.g. HIV) [53]. Another PS4≡ target, alpha 2-HS-glycoprotein (AHSG), was found to associate with the 3'-untranslated region of the HCV genome, potentially regulating HCV replication [54]. The PS4≡ profiling, in addition, allowed us to observe an increased activity of this protein, which could be mediated by HCV replication in Huh-7 cells (Figure 4, spot 7), suggesting a synergistic effect. Similarly, ETFA, involved in the beta-oxidation of fatty acids and the subsequent transfer of their electrons to the electron transport chain of the inner mitochondrial membrane, demonstrated a decreased activity during HCV replication (Figure 4, spot 19). Hepatitis C virus had been shown to inhibit mitochondrial electron transport and to increase reactive oxygen species (ROS) *in vitro *and *in vivo *[55], which correlates with the observed PS4≡ labeling decrease of ETFA. Furthermore, as this enzyme is part of the fatty acid catabolism (i.e. beta-oxidation), its decreased activity during HCV replication could constitute an additional contributing factor to abundant Huh-7 lipid accumulation and potentially be a cause in HCV-mediated steatosis. To the best of our knowledge, the remaining differentially active proteins identified by PS4≡ during HCV replication, such as protein disulfide isomerase A5 and nuclear distribution gene C homolog, have not been studied so far in context with HCV thus making the PS4≡ non-directed probe an important tool in identifying novel host-pathogen interactions. Further characterization of these enzymes will be required to confirm their differential activities and reveal their potential roles during HCV replication and infectivity.

## Conclusions

*In situ *ABPP using a PS4≡ probe identified 13 novel targets in Huh-7 cells replicating HCV RNA. Some, like HSP70A8, have a known importance in HCV propagation [44]. Six additional PS4≡ protein targets were previously identified in Huh-7 cells with a directed amino acid coupled quinolimine methide probe *in vitro *[19] validating this study's findings and methodology. Three novel potentially important host protein activity changes in the presence of replicating HCV involved proteins ETFA, protein disulfide isomerase A5, and nuclear distribution gene C homolog. These findings suggest that using both combinatorial and directed methods, either separately or in combination, can accelerate the discovery of protein activities associated with pathogenic states during HCV replication and infection. Collectively, these results highlight that *in situ *ABPP facilitates the probing of activity-based protein profiles that are predominantly or exclusively labeled in living cells. Furthermore, the strategy allows for the rapid identification of differentially active enzymes during HCV replication.

## Methods

### Chemical synthesis of PS4≡ probe, RhN_3_, and TBTA ligand

Synthesis of the PS4≡ probe, RhN_3_, and tris-(benzyltriazoylmethyl) amine (TBTA) ligand were performed as described previously [28,56].

### Tissue culture and reagents

The human hepatoma cell line Huh-7 was grown in monolayers with DMEM medium supplemented with 100 nM nonessential amino acids, 50 U/mL penicillin, 50 μg/mL streptomycin, and 10% FBS (CANSERA, Rexdale, ON). The Huh-7 cells stably expressing the subgenomic replicons (Figure 1A) (genotype 1B, con1) (passage number between 40 and 50) were maintained in the same culture medium supplemented with 250 μg/mL G418 Geneticin (GIBCO-BRL, Burlington, ON). The pFK-I389neo/NS3-3'/5.1 subgenomic replicon (Figure 1A) was kindly provided by Ralf Bartenschlager (Institute of Hygiene, University of Heidelberg, Germany).

### Active proteome extraction and *in vitro *activity based profiling

Subconfluent cells (80-90%) were washed and pooled with ice-cold 10 mM sodium phosphate buffer, pH 7. The cells were then subjected to Dounce-homogenization at 30% power (T8 Ika-Werke homogenizer, GMBH, Staufen, Germany) and sonicated (20 pulses, 50% duty cycle, Sonifier 250, Branson Ultrasonic, Danbury, CT) in ice-cold sodium phosphate buffer supplemented with 1% Triton X-100. The crude proteome extract was cleared by ultra centrifugation at 100,000 g, 4°C for 45 min, quantified with the BCA protein assay (Pierce, Rockford, IL) and diluted with sodium phosphate buffer to a final protein concentration of 1 mg/mL. The *in vitro *active proteome labeling conditions were optimized as follows (Figure 2A): the proteome extract (1 mg/mL in sodium phosphate buffer) was incubated in the dark with 20 μM of PS4≡ probe (from a 100 mM stock in dimethyl sulfoxide (DMSO), final DMSO concentration of 0.02%) for 1 h at 37°C with occasional mixing. After one hour, the reaction was quenched by precipitating the proteome with acetone: five volumes of ice-cold acetone were added to the sample, then the samples were frozen at -80°C for 30 min and subsequently centrifuged at 15,000 g, 4°C for 15 min to remove salts and unreacted probe.

### *In situ *PS4≡ protein profiling in Huh-7 hepatoma cells

The *in situ *active proteome labeling conditions were optimized (Figure 2B) to the following: the cell culture media of subconfluent cells (80-90%) was replaced with fresh media containing 100 μM PS4≡ probe (from a 100 mM stock in DMSO, final DMSO concentration of 0.1%) for 1 hr, 37°C, 5% CO2 in a cell culture incubator to allow for cellular uptake and proteome labeling. The cells were subsequently washed twice with ice-cold 10 mM sodium phosphate buffer, pH 7, and the proteome was harvested by Dounce-homogenization and sonication as described above.

### Bioorthogonal conjugation to RhN_3 _via cycloaddition reaction

*In vitro or in situ *PS4≡ labeled proteomes precipitated with acetone were resuspended in 200 μL distilled water. Cycloaddition reagents were added sequentially: 200 μM RhN_3 _(2.6 mM stock in DMSO), 2 mM sodium ascorbate (26 mM stock in water), and 200 μM TBTA ligand (2.6 mM stock in 1:4 DMSO:t-butanol). Prior to addition of the copper catalyst, the reaction mixture was vortexed, and 2 mM CuSO_4_.5H_2_O (26 mM stock in water) was added. Samples were mixed and incubated at 25°C for 1 hour in the dark at room temperature. The reaction was quenched by acetone precipitation as described above.

### 2D analysis of labeled proteomes

Precipitated protein pellets (200 μg) were resuspended in 125 μl of isoelectric focusing (IEF) buffer (7 M Urea, 2 M thiourea, 4% CHAPS, 1% DTT) containing 0.2% Ampholytes pH 3-10 (biolytes, Bio-Rad, Hercules, CA). IPG strips pH 3-10 NL, 7 cm (Bio-Rad) were passively rehydrated with the resuspended proteomes for 16 h. The rehydrated protein IPG strips were then submitted to isoelectric focusing using the Protean IEF Cell (Bio-Rad) with the following conditions: 200 V rapid ramp for 30 min, 500 V rapid ramp for 30 min, 2 h linear ramp to 6,500 V, and 2 h focusing (rapid ramp) at 6,500 V. The accumulated voltage-hour (VH) felt between a range of 12,000 - 17,500 VH.

Prior to SDS-PAGE separation, proteins immobilized on the IPG strips were reduced (1% DTT) and alkylated (4% iodoacetamide) in SDS equilibration buffer (6 M urea, 30% glycerol, 2% SDS, 50 mM tris-HCl, pH 8.8). The SDS-PAGE separation was performed on a 10% gel (1.5 mm thickness) at constant 150 V per gel. The gels were then scanned for fluorescence using the Typhoon 9410 (GE Healthcare, Piscataway, NJ) and were subsequently incubated overnight in fixing solution (50% v/v Ethanol, 5% v/v acetic acid). Finally, gels were stained with silver nitrate and scanned using the Fluor-S imager (Bio-Rad) in order to visualize the entire cellular proteome.

### Identification of labeled protein candidates

The fluorescent gel image was aligned with the silver stained gel image to locate the labeled protein spots. Protein candidates displaying heat-sensitive activity were selected as specific targets, while candidates showing heat-insensitive reactivity were considered as non-specific targets. The selected spots were manually excised under a laminar flow hood. Gels spots were destained with potassium ferricyanide solution (15 mM potassium ferricyanide, 50 mM sodium thiosulfate), rinsed three times with water, and then shrunk with acetonitrile. The gel pieces were reswelled with 20 μl of trypsin solution (0.01 μg/μl in 50 mM ammonium bicarbonate) and incubated overnight at 37°C.

Peptides were identified by LC-MS/MS on a LTQ XL (Thermo) mass spectrometer. The LTQ XL mass spectrometer (Thermo) was coupled to a MDLC chromatography system (GE Healthcare). The samples were first injected onto a 0.3 × 5 mm C18 micro pre-column cartridge (Dionex/LC Packings) to remove salts and other soluble contaminants. Samples were then separated on a 5 cm × 75 μm BioBasic C18, 5 μm particle, PicoFrit column (New Objective) with a flow rate of ~300 nL/minute using a 30 minute gradient: 0-30% acetonitrile/0.1% formic acid over 14 minutes, 30-50% acetonitrile/0.1% formic acid over 14 minutes, 50-90% acetonitrile/0.1% formic acid over 2 minutes. MS and MS/MS data were collected in enhanced profile and normal centroid mode (scan rate: 8,000 amu/sec, scan range: 400-1,600), respectively. MS/MS was performed on the three most abundant multiple-charged peaks for each MS scan.

Peak lists were generated using the default parameters by Extract-MSN in BioWorksBrowser 3.2 (ThermoFisher). LC-MS/MS spectra were searched against the National Centre for Biotechnology Information non-redundant database (October 16, 2006; 2,879,860 sequence entries; 1,012,985,077 residues) using Mascot daemon version 2.0 (Matrix Science, London, UK). Search parameters used for queries were trypsin cleavage, ≤1 missed cleavages, ± 1.5-Da peptide tolerance, ± 1.2-Da MS/MS tolerance, with no fixed modifications and the following variable modifications: carbamidomethyl (Cys), oxidation (Met). The results were then evaluated manually: protein identification required a MASCOT score above 50, a corresponding pI and mass according to their position after 2D gel electrophoresis, as well as being identified in at least three independent experiments.

### Northern analysis

Total RNA from subconfluent cells was isolated using the RNeasy extraction kit (Qiagen) and 1 μg was used to detect HCV replicon RNA and β-actin mRNA. Biotinylated negative sense probes complementary to HCV genome region 6648-7770 bp (NS5b) and β-actin (GenBank accession numbers AJ242654 and X00351, respectively) were synthesized using the MEGAscript T7 kit (Ambion, Foster City, CA), biotin-11-UTP and biotin-11-CTP (PerkinElmer, Boston, MA) following the supplier's protocol. Northern blotting and hybridization were performed using the NorthernMax kit (Ambion) and Hybond XL nylon membranes (Amersham Biosciences, Piscataway, NJ). The bound riboprobes were detected with the Chemiluminescent Nucleic Acid Detection Module (Pierce, Rockford, IL).

### Western immunoblotting

Proteomes (40 μg protein) were resuspended in SDS loading buffer, resolved by SDS-PAGE under reducing conditions (10% resolving gel), and transferred onto Hybond-P PVDF membrane (Amersham Biosciences). Thereafter, the membrane was blocked for 1 h in 5% dried skim milk in Tris-buffered saline (TBS) with 0.05% Tween-20. Human HSP70, PDIA1, AHSG, NUDC, ETFA, HCV NS5a, HCV NS3, and human β-tubulin were targeted with: a mouse monoclonal anti-HSP70 (1000-fold dilution in TBS-Tween; Abcam, Cambridge, UK), a mouse monoclonal anti-PDIA1 (5000-fold dilution in TBS-Tween, Abcam), a mouse monoclonal anti-human fetuin A/AHSG (200-fold dilution in TBS-Tween, R&D systems, Minneapolis, MN), a mouse monoclonal anti-NUDC (1000-fold dilution in TBS-Tween; Santa Cruz Biotechnology, Santa Cruz, CA), a rabbit polyclonal anti-ETFA (500-fold dilution in TBS-Tween; ProteinTech Group, Chicago, IL), a mouse monoclonal anti-NS5a (0.2 μg/mL in TBS-Tween; ViroStat, Portland, ME), a mouse monoclonal anti-NS3 (0.2 μg/mL in TBS-Tween; ViroStat), and a mouse monoclonal anti-β-tubulin (3.5 μg/mL in TBS-Tween; Sigma), respectively. After extensive washing, the membrane was incubated for 1 h with the donkey anti-rabbit IgG horseradish peroxidase (HRP)-conjugated antibody (0.26 μg/mL in TBS-Tween) or with the goat anti-mouse HRP-conjugated antibody (0.8 μg/mL in TBS-Tween; Jackson ImmunoResearch, West Grove, PA). Antigens were detected using enhanced chemiluminescence (ECL Western Blotting Detection Reagents; Amersham Biosciences).

### Statistical analysis

Individual experiments in this study were performed at least in triplicate in order to confirm the reproducibility of the results. Values are represented as means ± standard deviations. The statistical significance of differences between two or more means was evaluated by using analysis of variance (ANOVA); P values of less than 0.05 (indicated by asterisks) were considered to be statistically significant.

## Abbreviations

2D-GE: 2D-gel electrophoresis; ABPP: activity-based protein profiling; ANOVA: analysis of variance; AHSG: alpha 2-HS-glycoprotein; Cys: carbamidomethyl; DMSO: dimethyl sulfoxide; ETFA: electron transfer flavoprotein alpha polypeptide; HCV: hepatitis C virus; HSP70: Heat shock protein 70 kDa; IEF: isoelectric focusing; LD: lipid droplet; MudPIT: multidimensional protein identification technology; NS: non-structural; nu: nucleophile; NUDC: nuclear distribution gene C homolog; PDI: protein disulfide isomerise; PS4≡: phenyl sulfonate ester; RhN_3_: rhodamine azide; ROS: reactive oxygen species; TBTA: tris-(benzyltriazoylmethyl) amine; VH: voltage-hour.

## Competing interests

The authors declare that they have no competing interests.

## Authors' contributions

RS carried out the activity-based proteome analysis and the acquisition of the proteomic sequences. DRB participated in the design and in the coordination of the study, carried the analysis and interpretation of the proteomic sequences, and drafted the manuscript. CSM synthesized the phenyl sulfonate ester probe and reporter tag azide. JPP conceived, participated in the design and in the coordination of the study, and drafted the manuscript. All authors read and approved the final manuscript.
